# Comparing and Assessing Clinical Practice Guidelines and Consensus Statements for Healthy Athletes: A Scoping Review Protocol

**DOI:** 10.1002/gin2.70050

**Published:** 2025-10-29

**Authors:** Samyan Shahid, Andres Estupiñán‐Bohórquez

**Affiliations:** ^1^ Faculty of Sciences, McMaster University Hamilton Ontario Canada; ^2^ Institute of Medical Research, School of Medicine University of Antioquia Medellín Antioquia Colombia

**Keywords:** clinical practice guidelines, consensus statements, healthy athletes, methodological quality, sports medicine

## Abstract

**Introduction:**

Healthy athletes have unique physiological, psychological, and interpersonal needs that necessitate individualized prevention and management strategies. As such, this comprehensive protocol has been created to illustrate the scope, quality and coverage of these clinical practice guidelines (CPGs) and consensus statements among this specific population.

**Methods and Analysis:**

Searches will be conducted in PubMed, Scopus, SPORTDiscus, Web of Science, EMBASE, Google Scholar and any grey literature within official international organizations. Eligible publications will include CPGs and consensus statements that are aimed towards healthy athletes, published in English or Spanish since 2000. The AGREE II instrument will be applied to assess the quality of the guidelines by two independent reviewers. Disagreements will be resolved by a third reviewer. We will use descriptive statistics to summarize the main characteristics of the guidelines.

**Discussion:**

This scoping review seeks towards providing an in‐depth analysis on the mapping, explaining, and evaluating of the available CPGs and consensus statements of the population of healthy athletes. Moreover, it will identify thematic trends, methodological strengths, and potential evidence gaps that currently exist. Fundamentally, the results will provide valuable information towards development of guidelines, standardization efforts, and evidence‐based practice in the future within the field of sports medicine.

## Summary

- This scoping review will critically map, characterize, and evaluate the clinical practice guidelines (CPGs) and consensus statements that have been designed for the healthy athletes from all competitive levels (youth, collegiate, elite, recreational and master levels).
- The AGREE II instrument will be used during the evaluation to provide an assessment on the methodological rigour, transparency, and rigour of the documents included.
- There is a need for evidence, in which the findings will identify the thematic trends, methodological advantages, and any research gaps. This will benefit and shape the future guidelines and standards for sports medicine.
- The finding will help to enhance clinical decision making, new recommendations built upon high‐quality methods, and policy development. With regard to findings, it will support harmonization efforts across several athlete focused sports disciplines and federations.

**Abbreviations**
ACL: anterior cruciate ligament
CPG: clinical practice guidelines
ECG: electrocardiogram
GPS: global positioning system
HPO: hypothalamic‐pituitary‐ovarian
RED‐S: relative energy deficiency in sport
STI: sexually transmitted infection
TUE: therapeutic use exemptions
WADA: World Anti‐Doping Agency

## 1 Introduction

Healthy athletes constitute a distinct clinical population characterized by complex physiological, psychological, and social demands that affect health, performance, and long‐term well‐being [1, 2, 3]. As such, each requires a unique preventive, diagnostic, and management approach within sports medicine practice. Within the past decades, the field of sports medicine has progressively evolved to generate evidence‐informed recommendations targeted to the specific needs of healthy athletes across competitive contexts, including, youth, collegiate, elite, recreational and master levels [4, 5, 6]. As a result, based on such recommendations, a wide array of normative documents—namely CPGs and consensus statements—have been formalized and disseminated by sports federations, medical associations, scientific societies, and governmental bodies to support evidence‐based decision‐making in these athletic populations [7, 8, 9, 10].

CPGs are systematically developed statements that include key recommendations. These are aimed at improving patient care, guided by a structured examination of data, along with an assessment of the benefits and drawbacks of various options [11]. On the contrary, consensus statements are not always based on systematic evidence assessments; however, they are created by expert panels to provide guidance in areas where data is scarce or constantly changing [12]. Although consensus statements may be required when evidence is lacking, having reliance on the advice of experts or majority vote on its own may reduce transparency. Moreover, this may also cause an increase in variability, and the chance of introducing bias to the project. Subsequently, about sports medicine, key thematic areas addressed in these documents include injury prevention, mental health, nutrition, return‐to‐play protocols, and training under extreme environmental conditions. However, despite the increasing availability of such resources, there is currently no comprehensive synthesis that critically examines their presence, thematic scope, methodological development, or scientific quality [13, 14, 15].

Consequently, this scoping review has been designed to systematically map, describe, and assess the available CPGs and consensus statements targeting healthy athletes. Scoping reviews are a type of knowledge synthesis that explores the breadth and depth of evidence on a given topic, and one of which clarifies key concepts and knowledge gaps, where evidence is heterogeneous or emerging [16]. Particular emphasis will be placed on appraising their methodological quality and identifying key research gaps in normative coverage [17, 18, 19]. The primary objective of this study is to identify and classify CPGs and consensus statements that target healthy athletes (youth, collegiate, elite, recreational and master levels). This also includes determining the thematic areas by these recommendations. Secondary objectives within this study include an assessment on their methodological quality using the Appraisal of Guidelines for Research and Evaluation II (AGREE II) instrument [8]. We will also identify any evidence gaps and under‐regulated areas.

## 2 Methodology

This protocol describes a scoping review to identify the available guidelines for healthy athletes. This report was prepared following the recommendations by the Preferred Reporting Items for Systematic Reviews and Meta‐Analyses for protocols (PRISMA‐P) [20]. The final report of this study will follow the recommendations by the Preferred PRISMA‐P extension for Scoping Reviews (PRISMA‐ScR) checklist [17]. Although PRISMA‐P was initially designed for systematic reviews, it is used here to promote methodological transparency in this scoping review protocol. The methods will be guided by the JBI scoping review methodology [21] and the methods for developing reviews of guidelines by Johnston et al. [22]. Lastly, this protocol will be made publicly available through the Open Science Framework (OSF) once finalized and approved.

### 2.1 Eligibility Criteria

#### 2.1.1 Population

We will include CPGs, consensus statements or official advice aimed exclusively at healthy athletes that include those within youth collegiate, elite recreational and master levels. Among these healthy athletes, only those actively engaged in sports and continue to train despite having non‐debilitating medical illnesses (ex. seasonal allergies, mild asthma, mild gastrointestinal issues, or well‐controlled dermatological conditions) will be included [23]. When evaluating healthy and clinical populations, only the publications that clearly define components and suggestions for healthy athletes will be taken into consideration [24]. Reviewers will carefully determine if the preliminary topic or focus of a document is relevant to healthy athletes. In cases of potential doubts, inclusion decisions will be performed by agreement among reviewers to ensure consistency with the target population. Finally, we will exclude materials that focus solely on clinical populations with chronic illnesses, disabilities, or ongoing treatment and clinical rehabilitation [23].

#### 2.1.2 Concept

Eligible documents will include those that have inferable methodology. This includes those that meet at least one of the following criteria: provide a clear rationale for specific recommendations, cite the literature review process accordingly, or describe structured consensus techniques [25, 26]. This provides transparency and reliability in the selection process. We will exclude nonnormative resources, including editorials, opinion articles, unstructured expert comments, and other types of grey literature that lack a proper recommendation framework, will be excluded. Finally, any publications that do not provide or cite a methodological foundation or lack supporting evidence will also be excluded [22].

#### 2.1.3 Context

Eligible documents are those issued by recognized national or international organizations, such as sports federations, Olympic committees, scientific societies, professional groups, or government bodies. Documents published from the year 2000 onwards will only be considered. The year 2000 was selected, as it was the time when the AGREE instrument became standardized into guideline development methodology, and as such follows rigour and transparency [8]. Consequently, all included documents must be in English or Spanish that must contain explicit or inferable methodological development processes [17, 20, 21]. This is restricted to these languages for the feasibility of both screening and evaluation, while still covering a large portion of guideline publications in sports medicine. Finally, we will include all documents from any geographic region and cultural backgrounds as long as they meet the criteria stated above.

#### 2.1.4 Sources of Information

As for the literature search, it will be conducted among several different databases, which include the following: PubMed, Scopus, SPORTDiscus, Web of Science (Core Collection), and EMBASE. Subsequently, grey literature will be explored via Google Scholar and comprehensively searching official websites of international organizations (ex., IOC, FIFA, ITF, NCAA, and other Olympic federations). Reference lists from relevant reviews and documents will be scanned, and the corresponding authors or institutions will be contacted for inaccessible material.

#### 2.1.5 Thematic Framework

The thematic framework below includes a clear illustration of the categories and subcategories of expected guidelines. The authors of the research team built this framework based on expert judgement, including prior clinical and academic experience, through an initial scoping of pertinent guidelines. Since there is no currently established taxonomy for classifying normative texts linked to healthy athletes, the mentioned categories, and their respective subcategories reflect the main areas commonly addressed in athlete‐focused normative guidance. As a result, this framework is structured to assist the gathering of findings while staying adaptable to emerging themes.

#### 2.1.6 Search Strategy

A thorough search strategy will be conducted, and this will be used through Medical Subject Headings (MeSH) terms and free‐text terms. Additionally, the search strategy will combine key terms, as illustrated in the Appendix.

The search strategy will be adjusted before the final review scoping review publication, as this will allow for inclusion of most up to date publications.

#### 2.1.7 Selection Process

Before the full extraction and screening process that takes place, reviewers will participate in a calibration phase with the eligibility criteria. This will be done for a random sample of 10 documents to ensure accuracy and consistency. In the selection process, Covidence will be used for assisting with the screening and reviewer cooperation. Two independent reviewers will apply the eligibility criteria to both titles and abstracts. Afterwards, full texts of potentially eligible studies will be retrieved and assessed. In the case of any discrepancies, they will be resolved immediately through discussion or third‐party adjudication [21].

#### 2.1.8 Data Extraction

A standardized data extraction matrix will be applied to maintain consistency and transparency throughout the review process. The following information will be extracted for each document: the main topic must be stated, the type of document (ex. guideline or consensus statement), the issuing organization, the year of publication and the target population. Moreover, information about the target population, the development methodology used, the stakeholder involvement, and key recommendations will all be gathered. Lastly, any declared conflict of interest, and statements regarding the level of evidence will be identified.

#### 2.1.9 Methodological Quality Assessment

For this study, the AGREE II instrument will be used for assessing the methodological quality of the included documents [8]. As such, this tool evaluates six different domains: scope and purpose, stakeholder involvement, rigour of development, clarity of presentation, applicability, and editorial independence. Across these domains, descriptive tables with a mean score and a range of values will be incorporated, with scores spanning from 0% to 100%. Additionally, there will be graphical displays (ex. heat maps), which will be displayed for variability across all domains, along with summaries providing methodological strengths and limitations. Lastly, two trained assessors will perform a rigorous and thorough analysis, ensuring the scoring of each document occurs independently. A third reviewer will determine any underlying discrepancies of ≥ 2 points in domain scores.

#### 2.1.10 Synthesis of Results

The selected documents will be organized based on thematic area, athlete category, geographical region, and methodological quality. Descriptive tables will be used to summarize essential document characteristics. More importantly, thematic coding, guided by a constructive deductive method informed by a predetermined framework will aid in the discovery of the recurring topics, and potential gaps. There will also be an incorporation of visual tools such as matrices or heat maps, which can be created to show how themes are distributed between guidelines types, sports disciplines, and certain population groups. Finally, these studies will be accompanied by a narrative synthesis, which will highlight global trends and future priorities in sports medicine guideline development.

#### 2.1.11 Expected Impact

This scoping review will provide a comprehensive overview of current clinical guidelines and consensus statements among healthy athletes. The findings will support identifying areas that are well‐covered by high‐quality normative guidance, alongside any critical gaps in regulation.

Subsequently, the results that are obtained will be beneficial towards clinical decision‐making, policy development, and future creation of guidelines. These guidelines will be tailored accordingly to the specific needs of athletic populations, providing findings that support harmonisation efforts across several sports disciplines and federations. Furthermore, this review will contribute towards optimizing the health, safety, and performance of athletes worldwide.

#### 2.1.12 Patient and Public Involvement

No patients or members of the public were involved in the design, conduct, and reporting within this protocol.

## 3 Discussion

**Figure 1:**
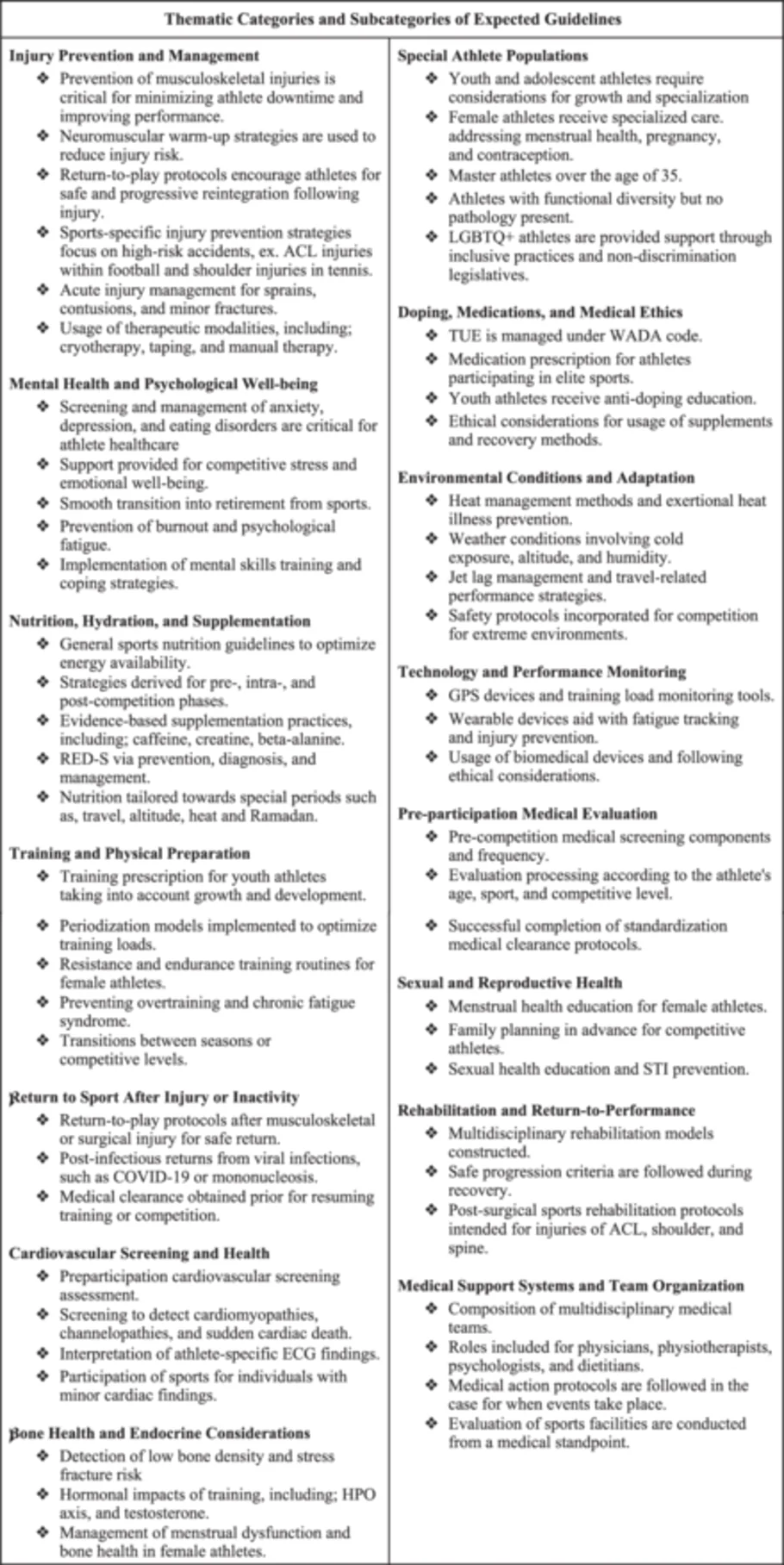
Thematic framework developed from initial scoping of included guidelines [27].

This scoping review will critically evaluate the scope, extent, and quality of CPGs and consensus statements, focusing specifically on healthy athletes across competitive levels (youth, collegiate, elite, recreational and master levels). As such, the evaluation will use the AGREE II instrument to assess the methodological rigour and transparency of such publications. Moreover, this method will also be used to identify recurring themes, underdeveloped fields, and normative gaps (Figure 1).

The findings will be useful to medical societies, policymakers, and sports organizations seeking to implement or improve athlete‐focused standards. Furthermore, this assessment will facilitate future CPG development by revealing methodological constraints, and content flaws among current publications. This guarantees that new recommendations are built upon high‐quality methods. As a result, the findings will assist clinicians in determining which high‐quality CPGs may be consistently adopted in the care of healthy athletes. Finally, this will also provide guidance to this growing field.

Within this scoping review, there are some anticipated limitations to be aware of. To begin with, limiting the possible documents to only English and Spanish may generate language bias. Alongside, restricting inclusion to documents published from 2000 limits the incorporation of relevant guidelines and CPGs in the sports medicine field. Nevertheless, due to the difficulty in accessing all necessary documents, there is a risk of publication bias, specifically with grey literature. There is also a possibility of potential overlap between guidelines and CPGs in similar themes, which may lead to redundancy, resulting in an analysis lacking comprehensiveness. Lastly, with the AGREE II instrument and scope of guidelines, there may be some involvement of subjectivity in the quality appraisal process, leading to possible misinterpretation of results. As such, recognizing such limitations beforehand provides contextualization of the data, and critical areas needed to be aware of.

## Author Contributions

Samyan Shahid led the conceptualization and drafting of the manuscript. Estupiñán‐Bohórquez provided methodological guidance, supervision, critical revisions, and approved the final version of the manuscript.

## Conflicts of Interest

The authors declare no conflicts of interest.

## Data Availability

This is a protocol, and as such no data was gathered or analysed. The final article derived from this study will be available following the journal's license requirements.

## Appendix A

*Search Strategy (In PubMedline)*:

(“athlete*” OR “sport*” OR “elite sports” OR “competitive sports”) AND (“guideline*” OR “consensus” OR “recommendation”) AND (“injury prevention” OR “hydration” OR “mental health” OR “nutrition” OR “returnto sport” OR “screening” OR “training” OR “retirement” OR “recovery” OR “environmental conditions” OR “menstrual health” OR “doping” OR “readaptation”).

*Search strategy in Embase*:

1. athlete*.mp.
2. sport*.mp.
3. 1 and 2
4. consensus.mp.
5. recommendation*.mp.
6. guidelines.mp. or practice guideline/
7. (practice adj guideline$).tw.
8. (clinical adj guideline$).tw.
9. (evidence adj2 recommendation$).mp.
10. or/4‐9
11. 3 and 10

*List of professional societies*:

1. International Olympic Committee (IOC) (https://www.olympics.com/ioc).
2. European College of Sport Science (ECSS) (https://sport-science.org/).
3. The American College of Sports Medicine (ACSM) (https://acsm.org/).
4. Canadian Society for Exercise Physiology (CSEP) (https://csep.ca/).
5. National Collegiate Athletic Association (NCAA) (https://www.ncaa.org/).
6. International Federation of Sports Medicine (FIMS) (https://www.fims.org/).
7. Australian College of Sport and Exercise Physicians (ACSEP) (https://www.acsep.org.au/).
8. British Journal of Sports Medicine (BJSM) (https://bjsm.bmj.com/).
9. American Medical Society for Sports Medicine (AMSSM) (https://www.amssm.org/).

## Appendix B

**Information to be extracted:**

1. Publication date.
2. Guideline authors.
3. Professional society.
4. Title of guideline.
5. Specific scope of guideline.
6. Target population of guideline.
7. Systematic review conducted (Yes/No).
8. GRADE methodology used (Yes/No)
9. Recommendations stated.
10. Strength of recommendations.
11. AGREE II Domain incorporated (1‐6).
